# Characterization of the HLA-DRβ1 third hypervariable region amino acid sequence according to charge and parental inheritance in systemic sclerosis

**DOI:** 10.1186/s13075-017-1253-9

**Published:** 2017-03-07

**Authors:** Coline A. Gentil, Hilary S. Gammill, Christine T. Luu, Maureen D. Mayes, Dan E. Furst, J. Lee Nelson

**Affiliations:** 1 Division of Clinical Research, Fred Hutchinson Cancer Research Center, 1100 Fairview Ave N, Seattle, WA 98109 USA; 2 Department of Obstetrics and Gynecology, University of Washington, Seattle, WA USA; 3 Division of Rheumatology and Clinical Immunogenetics, University of Texas Health Science Center at Houston, Houston, TX USA; 4 Division of Rheumatology, University of California, Los Angeles, CA USA; 5 Division of Rheumatology, University of Washington, Seattle, WA USA

**Keywords:** Systemic sclerosis, Human leukocyte antigen, Skewed parental inheritance

## Abstract

**Background:**

Specific HLA class II alleles are associated with systemic sclerosis (SSc) risk, clinical characteristics, and autoantibodies. HLA nomenclature initially developed with antibodies as typing reagents defining DRB1 allele groups. However, alleles from different DRB1 allele groups encode the same third hypervariable region (3rd HVR) sequence, the primary T-cell recognition site, and 3rd HVR charge differences can affect interactions with T cells. We considered 3rd HVR sequences (amino acids 67–74) irrespective of the allele group and analyzed parental inheritance considered according to the 3rd HVR charge, comparing SSc patients with controls.

**Methods:**

In total, 306 families (121 SSc and 185 controls) were HLA genotyped and parental HLA-haplotype origin was determined. Analysis was conducted according to DRβ1 3rd HVR sequence, charge, and parental inheritance.

**Results:**

The distribution of 3rd HVR sequences differed in SSc patients versus controls (*p* = 0.007), primarily due to an increase of specific DRB1*11 alleles, in accord with previous observations. The 3rd HVR sequences were next analyzed according to charge and parental inheritance. Paternal transmission of DRB1 alleles encoding a +2 charge 3rd HVR was significantly reduced in SSc patients compared with maternal transmission (*p* = 0.0003, corrected for analysis of four charge categories *p* = 0.001). To a lesser extent, paternal transmission was increased when charge was 0 (*p* = 0.021, corrected for multiple comparisons *p* = 0.084). In contrast, paternal versus maternal inheritance was similar in controls.

**Conclusions:**

SSc patients differed from controls when DRB1 alleles were categorized according to 3rd HVR sequences. Skewed parental inheritance was observed in SSc patients but not in controls when the DRβ1 3rd HVR was considered according to charge. These observations suggest that epigenetic modulation of HLA merits investigation in SSc.

**Electronic supplementary material:**

The online version of this article (doi:10.1186/s13075-017-1253-9) contains supplementary material, which is available to authorized users.

## Background

HLA class II genes contribute the largest portion of genetic risk for many autoimmune diseases including systemic sclerosis (SSc) [1]. The HLA DRB1 allele group DRB1*11 has been described consistently in association with SSc in Caucasian adults [1–3]. HLA nomenclature evolved utilizing serological typing reagents resulting in a “language of HLA”, with initial groupings based primarily on antibody recognition of the HLA molecule. However, the β1 chain of the HLA-DR molecule encoded by HLA-DRB1 is characterized by three regions of amino acid sequence hypervariability and the third hypervariable region (3rd HVR) is thought to be the most important site for T-cell recognition [4].

The 3rd HVR comprises amino acids 67–74 on the alpha helix of the HLA β1 chain. Allelic variation of the DRB1 DNA sequence can result in 3rd HVR amino acid sequences that differ for overall 3rd HVR charge and can impact the steric interaction between HLA molecules and T cells. The importance of charge in this region has been well demonstrated in rheumatoid arthritis, where different risk-associated DRβ1 molecules carry 3rd HVR sequences with a positive charge, whereas a negatively charged 3rd HVR sequence is associated with protection from the disease [5, 6].

In the current study we investigated the DRβ1 3rd HVR, charge, and parental transmission in SSc patients and healthy controls. We sought to examine inheritance according to parental origin because biased HLA transmission has been described in some other autoimmune diseases, including multiple sclerosis, and in some but not all studies of type 1 diabetes [7–9]. The primary purpose of this study was to test the hypothesis that SSc risk is modulated according to whether the mother or the father transmitted an HLA-DRB1 allele and is impacted by DRβ1 3rd HVR charge.

## Methods

### Study participants

A total of 306 unrelated families were studied, 121 in which the proband had SSc and 185 healthy control families. SSc patients were recruited primarily from the Seattle, WA, USA area, with some patients from Alaska, Montana, Oregon and other states and some identified through the SSc family registry based in Houston, TX, USA. Healthy controls were recruited primarily from the Seattle, WA area. The median age at the time of sample collection for HLA genotyping for SSc patients was 43 years (range 18–62) and for controls was 31 years (range 4–73). SSc patients were female and controls were predominantly female (99 female, 22 male). All patients and controls were Caucasian. Study subjects were included providing maternal versus paternal transmission of HLA-DRB1 could be determined by HLA genotyping studies of the patient or control and family members. Eight SSc patients and one control were HLA genotyped but not included in the analysis because parental haplotype transmission could not be determined. All subjects provided written informed consent. The study was approved by the Institutional Review Board of Fred Hutchinson Cancer Research Center.

### HLA genotyping

HLA genotyping was conducted from peripheral blood, buccal swabs, or mouthwash specimens. Genomic DNA was extracted from whole blood or peripheral blood mononuclear cells using the Wizard Genomic DNA Purification Kit (Promega, Madison, WI, USA), from mouthwash specimens using the High Pure PCR Template Preparation Kit (Roche Diagnostics, Indianapolis, IN, USA), or from buccal swabs using the BuccalAmp DNA Extraction Kit (Epicentre Biotechnologies, WI, USA). All subjects were genotyped for HLA-DRB1, as well as for DQA1 and DQB1 loci. DNA-based typing was conducted with the Luminex-based PCR-sequence-specific oligonucleotide probe technique (Luminex; One Lambda, Canoga Park, CA, USA) with alleles assigned using HLA Fusion™ 3.0 standard analysis software, or Dynal strip detection with sequence-specific oligonucleotide probes (Dynal RELITM SSO, UK) followed by identification of specific alleles by sequencing (Applied Biosystems, Foster city, CA, USA). Amino acid sequences were determined based on the specific alleles and the International Immunogenetics Information System for HLA (IGMT/HLA; www.ebi.ac.uk/ipd/imgt/hla/allele.html).

### HLA-DRβ1 3rd HVR classification

All DRB1 alleles were first categorized according to the encoded 3rd HVR amino acid sequence, from positions 67 through 74 of the HLA-DRβ1 chain (Table 1). Among all possible DRβ1 3rd HVR sequences, 17 were present in at least one study subject with eight sequences present in at least 5% of either patients or controls. Next, 3rd HVR sequences were grouped according to their overall charge: +2, +1, 0, −1, or −2.

**Table 1:** HLA-DRβ1 categorized according to 3rd HVR sequences, DRB1 alleles encoding each sequence, and associated charges

3rd HVR	aa 67–74	DRB1 alleles	Charge
1	LLEQRRAA	01:01, 01:02, 01:08, 04:04, 04:05, 04:08, 14:02, 14:06, 14:20	+1
2	I--DE---	01:03, 04:02, 11:02, 13:01, 13:02, 13:04, 13:28	−2
3	---R----	10:01	+2
4	I---A---	15:01, 15:02, 15:03	0
5	F--D----	11:01, 11:04, 12:02, 13:05, 16:01	0
6	---D----	16:02	0
7	----K---	04:01	+1
8	-------E	04:03, 04:07	0
9	I--D--GQ	07:01	0
10	F--R---E	09:01	+1
11	F--D---L	08:01, 08:02, 08:04, 08:06	0
12	I--D---L	08:03	0
13	F--DE---	11:03	−2
14	I--D----	12:01	0
15	----K-GR	03:01	+2
16	I--DK---	13:03	0
17	---R---E	14:01	+1

Summary is for all 3rd HVR sequences and DRB1 alleles observed in at least one study subject

*3rd HVR* third hypervariable region, *aa* amino acids

In our patient and control populations no subject had a DRB1 allele encoding a 3rd HVR sequence with a −1 charge. Therefore, our study compares four charge groups between SSc and controls.

### Statistical analysis

After testing the normality of the distribution of continuous variables with the Shapiro–Wilk test, comparisons were made with the Wilcoxon rank-sum test as appropriate. Categorical variables were compared utilizing chi-square tests or, when indicated, Fisher exact tests. The primary outcome analyzed was maternal versus paternal inheritance of the DRβ1 3rd HVR sequence according to charge. In addition, we compared the overall distribution of 3rd HVR sequences and 3rd HVR charge in SSc patients and controls. Analysis was conducted when 5% or more of patients or controls were represented in a category and correction made for multiple comparisons by Bonferroni adjustment of the threshold for significance. Logistic regression was carried out adjusting for sex because some controls were male, but this revealed no differences from the unadjusted associations.

Because a parent of origin effect has not been studied previously in SSc, a precise estimate of statistical power is precluded. However, our available sample size of 121 cases and 185 controls is estimated to provide more than 90% power to detect a two-fold difference in parental origin from an expected 50/50 in controls to 25/75 in cases, assuming α = 0.05 and a two-tailed test.

## Results

Among SSc patients, 64% had diffuse SSc and 36% limited SSc (77 and 44 respectively). Antibodies to topoisomerase I (ATA) were positive in 34% of patients (37 of 109 tested) and to centromere (ACA) in 19% of patients (17 of 90 tested). The median age of SSc onset was 37 years, range 16–58.

HLA-DRB1 alleles were classified based on the amino acid sequence of the encoded 3rd HVR (amino acids 67–74) and the overall charge of each unique 3rd HVR sequence was determined (Table 1). Comparing SSc patients with healthy controls, the overall distribution of 3rd HVR sequences was significantly different (*p* = 0.007) (Table 2). The sequence “FLEDRRAA” (sequence category 5 in Table 1) was significantly enriched in SSc patients compared with controls (*p* = 0.004, *p* value corrected for multiple comparisons *p*_c_ = 0.032), primarily due to enrichment of DRB1*11:04 among SSc patients, as reported previously [3]. The sequence “FLEDRRAL” (sequence category 11 in Table 1) was also enriched in SSc patients (*p* = 0.006, *p*_c_ = 0.048) (Table 2), due to some increase of DRB1*08:01 and DRB1*08:02 in SSc patients. These observations are concordant with a previous report of an increase in the motif “FLEDR” in a French population [10]. In contrast to the French study, however, we did not see enrichment of this sequence encoded by DRB5, which instead was decreased in our population (data not shown).

**Table 2:** Allele frequencies classified according to the third hypervariable region in controls and SSc patients

	Controls	SSc		
HVR region	*n* (%)	*n* (%)	*p* value	OR (95% CI)
1	68 (18.4)	34 (14.0)	0.160	0.73 (0.46–1.14)
2	44 (11.9)	24 (9.9)	0.447	0.82 (0.48–1.38)
3	3 (0.8)	1 (0.4)		
4	58 (15.7)	21 (8.7)	0.012	0.51 (0.30–0.87)
5	36 (9.7)	43 (17.8)	0.004^§^	2.00 (1.25–3.23)
6	0 (0.0)	1 (0.4)		–
7	34 (9.2)	25 (10.3)	0.640	1.14 (0.66–1.96)
8	4 (1.1)	8 (3.3)		
9	48 (13.0)	29 (12.0)	0.718	0.91 (0.56–1.49)
10	4 (1.1)	2 (0.8)		
11	8 (2.1)	16 (6.6)	0.006^#^	3.20 (1.35–7.61)
12	1 (0.3)	0 (0.0)		–
13	5 (1.3)	6 (2.5)		
14	6 (1.6)	4 (1.7)		
15	41 (11.1)	26 (10.7)	0.896	0.97 (0.57–1.63)
16	3 (0.8)	0 (0.0)		–
17	7 (1.9)	2 (0.8)		
All	370	242		

Third HVR overall distribution, SSc patients versus controls, *p* = 0.007

^§^ *p*_c_ = 0.032

^#^ *p*_c_ = 0.048

*SSc* systemic sclerosis, *HVR* hypervariable region, *OR* odds ratio, *CI* confidence interval, p_*c*_ *p* value corrected for multiple comparisons

**Fig. 1:**
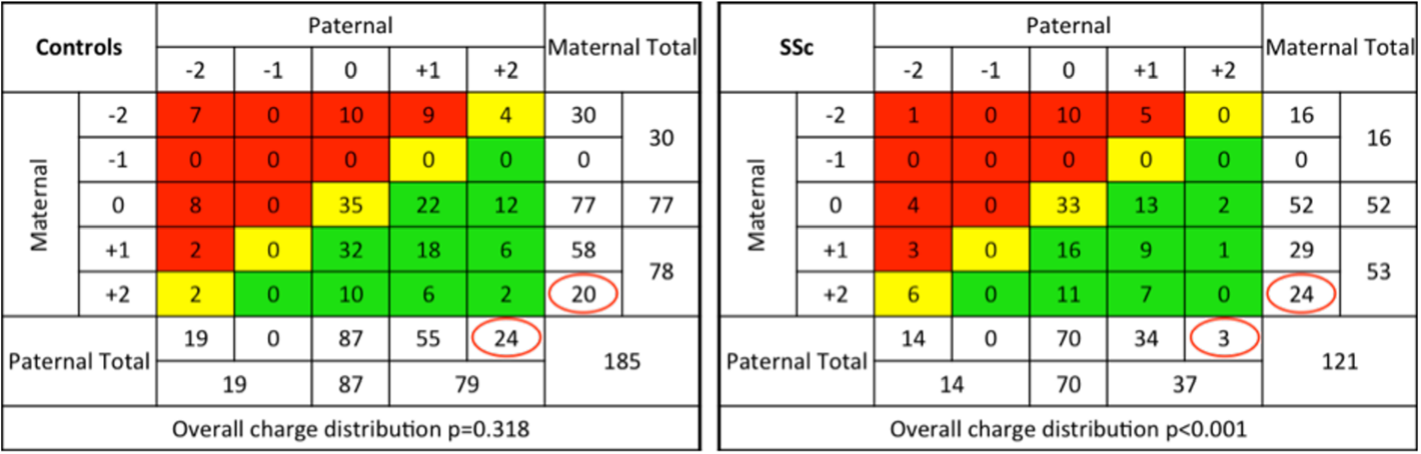
Distribution of HLA-DRβ1 3rd HVR sequences according to charge and parental inheritance. Colors represent the overall 3rd HVR charge for each (biallelic) individual, considering both maternal and paternal haplotypes; *red*, overall charge was negative; *yellow*, overall charge was neutral; and *green*, overall charge was positive. Among SSc patients there was marked skewing of parental inheritance when the 3rd HVR sequence carried a +2 charge (*p* = 0.0003, *p*_c_ = 0.001) whereas controls showed a similar frequency of inheritance from either parent, as would be expected. Skewing was also observed, to a lesser extent, among SSc patients when the 3rd HVR sequence carried a 0 charge (*p* = 0.021, *p*_c_ = 0.08). Columns and rows with a −1 charge are 0 because no study subject had a 3rd HVR sequence with this charge (Color figure online). *SSc* systemic sclerosis

Overall, the 3rd HVR charge on SSc haplotypes was similar to that of controls; of the 242 SSc and 370 control haplotypes respectively, 11% and 12% had a +2 charge, 26% and 31% had a +1 charge, 50% and 44% had a charge of 0, and 13% and 13% had a −2 charge. As noted previously, no 3rd HVR sequence represented in our populations carried a −1 charge. Thirty-six percent of SSc patients and 34% of controls carried the same charge on both parental haplotypes. When considering both haplotypes together, 19%, 32%, and 49% of the SSc patients had overall negative, neutral, or positive charge respectively, compared with 20%, 22%, and 58% of healthy controls (Fig. 1).

Analysis was conducted considering maternal or paternal DRB1 allele inheritance according to the charge of the DRβ1 3rd HVR. The overall distribution of inheritance indicated significant skewing among SSc patients (*p* < 0.001). Among SSc patients the father transmitted a 3rd HVR sequence with a +2 charge significantly less often than the mother (*p* = 0.0003). Almost 90% of the time, transmission was maternal rather than paternal (Fig. 1). This skewed parental inheritance remained significant (*p* = 0.001) when corrected for comparing four different categories of charge. The skewed inheritance was observed among patients with both diffuse and limited SSc and was not different for those with younger versus older age of SSc onset. Skewed inheritance for overall distribution, and for each charge category, was not observed in the control population in which inheritance was not different from 50–50, as expected. To a lesser extent, 3rd HVR sequences with a 0 charge were inherited more often from the father than the mother among SSc patients (*p* = 0.021, *p*_c_ = 0.084). Parental transmissions of 3rd HVR sequences with +1 or −2 charge were not significantly skewed (Fig. 1).

While our study was designed to test a hypothesis regarding charge of the DRβ1 3rd HVR sequence and parental inheritance, linkage disequilibrium of DRB1 is very strong with DQA1 and DQB1, and secondary analysis was conducted for these loci. DQβ1 has a similarly located hypervariable region from amino acids 70 through 77. DQB1 alleles represented in our study populations had an overall net charge of +3, +1, 0, −1, or −2 (Table 3). Not surprisingly, the +3 charge for DQβ1 was also significantly decreased for paternal versus maternal transmission, because DRB1*03:01 (+2 DRβ1 3rd HVR) haplotypes in our populations with few exceptions carried DQB1*02:01, which has a +3 charge. Patterns of linkage disequilibrium did not permit clear separation of the role of DQB1 from DRB1. However, the skewing for DQβ1 with a +3 charge was less pronounced than that described for DRβ1; eight were transmitted paternally versus 31 transmitted maternally. Additionally, DRB1*07:01 haplotypes can carry either DQB1*02:02 (+3) or DQB1*03:03 (0), and there was no suggestion of skewed paternal inheritance for these two haplotypes or an increase in DRB1*07:01–DQB1*02:02 haplotypes in the SSc population overall (Additional file 1: Table S1).

**Table 3:** DQβ1 hypervariable region and charge, amino acids 70–77^a^

	Amino acid position								Overall charge
Allele	70	71	72	73	74	75	76	77	
05:01/2/3	G	A	R	A	S	V	D	R	+1
06:01/4/9/11, 03:01/2/3	**R**	T	**–**	**–**	**E**	**–**	**–**	**T**	0
06:02/3/11/16	**–**	T	**–**	**–**	**E**	L	**–**	**T**	−1
02:01/2	**R**	**K**	**–**	**–**	A	**–**	**–**	**–**	+3^b^
04:01/2	**E**	**D**	**–**	**–**	**–**	**–**	**–**	**T**	−2

^a^Amino acids that differ from the consensus (alleles on the first line) by charge are presented in bold. Sequences encoded by uncommon alleles not present in our study populations are not included

^b^Among SSc patients with DQβ1 + 3 charge, eight were inherited paternally and 31 inherited maternally (*p* corrected = 0.001)

Polymorphisms on DQα1 are distributed somewhat differently from DRβ1 and DQβ1, but considered for amino acids 47–56 the net charge was +4, +2, or +1 for alleles represented in our study population and did not differ due to parental inheritance (Table 4). This result was also consistent with linkage disequilibrium in the HLA class II region, but results for DQA1 could be distinguished from DRB1 because multiple different DRB1 alleles are on haplotypes with the similar DQA1 alleles and some DQA1 alleles other than DQA1*05:01 also encode a +2 charge.

**Table 4:** DQα1 hypervariable region and charge, amino acids 47–56^a^

	Amino acid position										Overall charge^b^
Allele	47	48	49	50	51	52	53	54	55	56	
01:01/2/3/4/5	R	W	P	E	F	S	K	F	G	G	+1
02:01	K	**–**	**–**	**L**	**–**	**H**	R	**–**	**R**	**–**	+4
03:01/2/3	**Q**	**–**	**–**	**L**	**–**	**R**	R	**–**	**R**	**R**	+4
04:01	**C**	**–**	**–**	**V**	**–**	**R**	**Q**	**–**	**R**	**–**	+2
05:01/3/5	**C**	**–**	**–**	**V**	**–**	**R**	**Q**	**–**	**R**	**–**	+2
06:01	**C**	**–**	**–**	**V**	**–**	**R**	**Q**	**–**	**R**	**–**	+2

^a^Amino acids that differ from the consensus (alleles on the first line) by charge are presented in bold. Sequences encoded by uncommon alleles not present in our study populations are not included

^b^Parental inheritance was not skewed according to any DQα1 charge category. Similarly, results were not skewed if alternatively considered to extend to include a polymorphism at DQα1 amino acid 64

As expected because of linkage disequilibrium, the DRB1*03:01–DQA1*05:01–DQB1*02:01 haplotype was similarly skewed with three inherited paternally versus 21 inherited maternally (*p*_c_ = 0.001), although again skewing was somewhat less pronounced than when considered for DRβ1. (Haplotypes that were common in our study population are presented in Additional file 1: Table S1.)

## Discussion

In the current study we investigated patients with SSc and healthy controls, analyzing the DRβ1 3rd HVR according to charge irrespective of specific DRB1 alleles and considered parental inheritance. The importance of the DRβ1 3rd HVR and of charge in this region is well established in another autoimmune disease, rheumatoid arthritis, for which underlying HLA disease susceptibility is believed to be due to amino acid motifs of the DRβ1 3rd HVR that carry a similar charge [5, 6]. Our primary interest was to ask whether SSc patients differed from healthy controls if considered for parental origin of the DRβ1 3rd HVR categorized according to charge. In contrast to healthy individuals among whom parental inheritance was 50–50 as expected, we found significant skewing of parental inheritance in SSc patients. The most striking finding was that the father transmitted a DRB1 allele encoding a +2 charge 3rd HVR significantly less often than the mother in SSc patients. With respect to DRB1 allele frequencies, our study population was similar to prior reports of similar populations [1–3], with the patient population primarily enriched for DRB1*11:04 compared with controls.

Skewed parental inheritance of HLA-DRB1 alleles in SSc strongly suggests a parent-of-origin effect. Parent-of-origin effects are well described for a number of genes and are especially well recognized as important in early development [11]. Skewing from the expected random (50–50) inactivation of maternal versus paternal X-chromosomes has been reported previously in women with SSc, as well as in women with rheumatoid arthritis and some other diseases [12, 13]. A few studies have evaluated parental inheritance of HLA in other autoimmune diseases. In type 1 diabetes, parent-of-origin skewing in HLA inheritance has been described in some, but not all, studies [8, 9]. In patients with multiple sclerosis, HLA transmission was distorted by the parent of origin and by gender of the affected offspring [7]. A differential methylation signal with a peak at HLA-DRB1 was observed in CD4^+^ T cells of multiple sclerosis patients compared with controls in another study, suggesting a potential explanation for this observation [14].

A number of phenomena can underlie a parent-of-origin effect. The best characterized parent-of-origin effect is that associated with genomic imprinting [11]. Embryonic lethality is one explanation for a parent-of-origin effect and, although skewing was not observed in our healthy population, it is possible that an extreme phenotype could result in loss of embryos destined to develop SSc in later life. The rate of decay for small molecules or epigenetic marks in sperm and ova affecting survival to gametogenesis has also been hypothesized to play a role in distorted haplotype transmission [7]. Additionally, the molecules CCCTC-binding factor (CTCF) and Class II transactivator (CIITA), two units of a transcription complex involved in HLA class II gene transcription, are thought to be subject to epigenetic modifications [15, 16]. The current results are of further interest in light of a recent study that implicated fetal programming in SSc [17] as well as increasing evidence for a role of epigenetic regulation in SSc pathogenesis [18].

There are a number of limitations to our study. SSc patients in our study were all female and it would be of additional interest to know whether males and/or children with SSc differ. Some of our controls were male and younger than the patient population. However, neither would be anticipated to affect the overall study observations because controls had the expected random (50–50) distribution regardless of gender or age. SSc is also a rare disorder offsetting any potential bias due to development of SSc later in life by a younger control. Another limitation is that all of our study subjects were Caucasian because too few patients from other racial/ethnic backgrounds were available for analysis. Also, the number of SSc patients was modest and a larger study would be needed to evaluate potential differences according to clinical and autoantibody characteristics. Another limitation is that alleles carrying a +2 charge other than DRB1*03:01 were uncommon in our population so the skewed inheritance observed could be specific to genes on the DRB1*03:01 haplotype, including DQB1*0201, rather than the DRβ1 3rd HVR +2 charge. It should also be added that some amino acids are subject to posttranslational modification that results in a change of charge [19] and, while this has largely been examined on autoantigens, HLA molecules are themselves also presented as self-peptides by other HLA molecules [20].

## Conclusions

Skewed parental HLA inheritance was observed among SSc patients compared with healthy controls. Paternal transmission was significantly reduced compared with maternal transmission when the HLA-DRB1 allele encoded a 3rd HVR with a +2 charge and to a lesser extent increased when charge was 0, in SSc patients but not in controls. To our knowledge, skewed parental inheritance of HLA-DRB1 alleles has not been reported previously in SSc. Provided our results are replicated, future investigations into the underlying mechanism(s) for a parent-of-origin effect of HLA inheritance in SSc may lead to new insight into the role of HLA molecules in SSc pathogenesis.

## Additional file

Additional file 1: Table S1. DRB1-DQA1-DQB1 common haplotypes in SSc patients and controls. (DOCX 44 kb)

## Abbreviations

HLA: Human leukocyte antigen (or allele)
HVR: Hypervariable region
SSc: Systemic sclerosis
