# Supplementary material for: Characterization of the HLA-DRβ1 third hypervariable region amino acid sequence according to charge and parental inheritance in systemic sclerosis
Source: Arthritis Res Ther. 2017 Mar 7;19:46. doi: 10.1186/s13075-017-1253-9 (PMC5341397; doi:10.1186/s13075-017-1253-9)
Supplement: Additional file 1: Table S1. — DRB1-DQA1-DQB1 common haplotypes in SSc patients and controls. (DOCX 44 kb) [file 13075_2017_1253_MOESM1_ESM.docx]

**Additional file 1: Table S1. DRB1-DQA1-DQB1 common haplotypes, SSc and Controls**^

SSc N=121 Controls N=185

DRB1 DQA1 DQB1 # % # %

01:01 01:01 05:01 17 14.0% 44 23.8%

15:01 01:02 06:02 15 12.4% 54 29.2%

03:01 05:01 02:01 24 19.8% 41 22.2% ^§^

04:01 03:01/03 03:01/02 23 19.0% 34 18.4%

11:01 05:05 03:01 12 9.9% 21 11.3%

11:04 05:05 03:01 21 17.4% 5 2.7%

13:01 01:03 06:03 10 8.3% 15 8.1%

13:02 01:02 06:04 4 3.3% 12 6.5%

07:01 02:01 02:02 14 11.6% 33 17.8%

07:01 02:01 03:03 14 11.6% 14 7.6%

^ Haplotypes present in at least 5% of SSc patients or controls

^§^ The DRB1*03:01-DQA1*05:01-DQB1*02:01 haplotype was not increased overall, however, parental inheritance was skewed among SSc patients with 3 paternally versus 21 maternally inherited (p corrected=0.001). No other haplotype was significantly skewed for parental inheritance.
